# Torsion Pendulum Apparatus for Ground Testing of Space Inertial Sensor

**DOI:** 10.3390/s24237816

**Published:** 2024-12-06

**Authors:** Shaoxin Wang, Zuolei Wang, Dongxu Liu, Peng Dong, Jian Min, Ziren Luo, Keqi Qi, Jungang Lei

**Affiliations:** 1 Center for Gravitational Wave Experiment, National Microgravity Laboratory, Institute of Mechanics, Chinese Academy of Sciences (CAS), Beijing 100190, China; wangshaoxin@imech.ac.cn (S.W.);; 2 Taiji Laboratory for Gravitational Wave Universe (Beijing/Hangzhou), University of Chinese Academy of Sciences (UCAS), Beijing 100049, China; 3 Space Environmental Load Engineering Center, Lanzhou Institute of Physics, Lanzhou 730000, China; wzlgam2000@163.com (Z.W.);; 4 Department of Modern Mechanics, School of Engineering Science, University of Science and Technology of China, Hefei 230026, China; 5 Hangzhou Institute for Advanced Study, University of Chinese Academy of Sciences, Hangzhou 310024, China

**Keywords:** torsion pendulum, inertial sensor, test mass, sensitivity

## Abstract

The precise movement of the test mass along a geodesic is crucial for gravitational wave detection in space. To maintain this motion, the core payload-inertial sensor incorporates multiple functional units designed to mitigate various sources of stray force noise affecting the test mass. Understanding the limits of these noise sources is essential for enhancing the inertial sensor system design. Additionally, thorough ground-based verification of these functional units is necessary to ensure their reliability for space missions. To address these challenges, we developed a low-frequency torsion pendulum apparatus that utilizes a commercial autocollimator as the optical readout element for testing this type of space inertial sensor. This paper provides a comprehensive overview of the apparatus’s operating principle, structural characteristics, and the results of laboratory tests of its background noise. Experimental data demonstrate that the torsion pendulum achieves a sensitivity of 1 × 10^−11^ Nm/Hz^1/2^ within the measurement band from 1 mHz to 0.1 Hz, confirming its suitability for various inertial sensor tests. Furthermore, the insights gained from constructing the torsion pendulum will inform future system upgrades.

## 1. Introduction

The ground-based Laser Interferometer Gravitational-Wave Observatory (LIGO) made its first direct detection of gravitational waves in 2015 [1], confirming Einstein’s century-old prediction from his general theory of relativity [2]. This breakthrough opened new avenues for observing the universe beyond electromagnetic waves [3].

In contrast to ground-based detectors, the space environment offers greater stability, enabling the construction of a laser interferometer with arms extending up to a million kilometers. This enhanced characteristic allows for the detection of weaker, lower-frequency gravitational wave signals below 1 Hz. The frequency band is home to a broader range of gravitational wave sources, providing a wider array of scientific targets and contributing to a deeper understanding of the universe [4]. These advantages led to the proposal of the Laser Interferometer Space Antenna (LISA) [5], which successfully verified key technologies through the LISA Pathfinder (LPF) project in 2015 and has now entered its final implementation phase [6,7].

Following the development of LISA, several other space-based programs were proposed to detect gravitational waves using laser interferometry. Notable programs include the Big Bang Observer (BBO) [8], the Deci-Hertz Interferometer Gravitational-Wave Observatory (DECIGO) [9], the Astro-dynamical Space Test of Relativity (ASTROD) [10], Taiji [11], and TianQin [12].

The Taiji program, China’s first space-based gravitational wave detection mission, was proposed by the Chinese Academy of Sciences in 2008. Employing a constellation configuration similar to LISA, Taiji aims to establish a laser interferometer with a baseline of 3 million kilometers in the Sun’s orbit to detect gravitational waves [13]. In 2019, Taiji launched the Taiji-1 technology verification satellite to test its critical technologies, including laser interferometer, inertial sensor (IS), and drag-free control systems [14].

In order to achieve the highly sensitive detection of low-frequency gravitational waves, the residual acceleration of the test mass (TM) moving along the geodesic orbit must be less than 3 × 10^−15^ m/s^2^/Hz^1/2^, from 0.1 mH to 1 Hz [15]. To meet this stringent requirement, space gravitational wave detection missions are equipped with ISs to maintain the ultra-precise motion of the TM. On the one hand, the IS can mitigate the impact of noise directly acting on the TM by employing a clever design and integrating various functional units [16]. On the other hand, ISs precisely measure and control the pose of the test mass using capacitive sensors. It also works in tandem with micro-thrusters to achieve the drag-free flight of the spacecraft, further reducing coupling noise from the spacecraft to the TM. Additionally, the inertial sensor must address other engineering challenges, making its structure complex. This complexity introduces higher risks for space missions, necessitating rigorous ground-based verification tests to ensure mission success [17,18].

The ground testing of ISs primarily focuses on accurately estimating the upper limit of noise and validating noise models. This is particularly crucial for identifying and mitigating the main noise sources that limit the sensitivity of space-based gravitational wave detectors, including magnetic fields, temperature gradients, residual gas Brownian motion, charge accumulation, and coupling effects. Since each type of noise is more than 10 orders of magnitude smaller than gravitational acceleration [19], specialized devices are required to achieve the necessary precision. The torsion pendulum, a classical weak force measurement system, is highly effective at isolating the influence of Earth’s gravitational field and sensitive to weak torques that can be converted to corresponding forces through suitable assumptions and models in the direction of torsion. It is widely used in various fundamental physics experiments and has become the preferred device for the ground testing of ISs [20,21,22]. Numerous research institutions worldwide have developed torsion pendulum systems, resulting in various structural designs. However, only a few space mission teams have conducted relevant research on ISs.

Among these, the University of Trento stands out for its comprehensive research, having developed multiple torsion pendulum systems tailored to different functional units of ISs. Their systems achieve a torque measurement resolution of 0.1 × 10^−15^ Nm/Hz^1/2^ at 1 mHz [23,24,25]. Other Italian research institutions, including the University of Rome and INFN, have jointly developed a two-DOF torsion pendulum to characterize the geodesic motion of the TM for free-fall space missions. This system is capable of the simultaneous measurement of both the acceleration and torque acting on the TM; it has a torque resolution of 6 × 10^−13^ Nm/Hz^1/2^ at 1 mHz [26,27]. The University of Florida has developed a functional integrated test system based on a polarization-multiplexed heterodyne interferometer with an acceleration resolution of 2 × 10^−12^ m/s^2^/Hz^1/2^ at 0.5 mHz [28,29]. Huazhong University of Science and Technology has also created a torsion pendulum system to evaluate accelerometer performance, utilizing the favorable conditions of an underground laboratory to achieve a torque resolution of 20 × 10^−15^ Nm/Hz^1/2^ at 1 mHz [30,31]. Additionally, the University of Washington and the Max Planck Institute for Gravitational Physics (Albert Einstein Institute) have developed similar apparatuses based on the laser interferometer readout system, achieving a resolution 2 × 10^−14^ Nm/Hz^1/2^ at 0.1 Hz and 1 × 10^−12^ Nm/Hz^1/2^ at 4 mHz, respectively [32,33].

In this study, we present our torsion pendulum system, specifically designed for the ground testing of ISs. Section 2 provides a detailed analysis of the factors limiting sensitivity, based on the fundamental principles of the torsion pendulum. Section 3 describes the system-level design in detail. In Section 4, we describe the entire system and a measurement experiment to assess background noise. The results indicate that the system achieves a torque resolution of 9.5 × 10^−12^ Nm/Hz^1/2^ below 0.1 Hz, which is converted into acceleration of about 1.1 × 10^−10^ m/s^2^/Hz^1/2^, an order of magnitude higher than the requirement of 3 × 10^−9^ m/s^2^/Hz^1/2^@10 mHz for low-orbit applications of the Taiji program at the present stage. Finally, Section 5 summarizes the device’s characteristics and discusses potential applications and future upgrades.

## 2. The Basic Principle of the Torsion Pendulum

As previously mentioned, a thorough, ground-based, experimental verification is required before launching an IS into space. Even for the pathfinder in the second phase of the Taiji program, the residual acceleration noise must be less than 3 × 10^−13^ m/s^2^/Hz^1/2^ [34], particularly at frequencies below 0.1 Hz, which presents a significant challenge.

**Figure 1:**
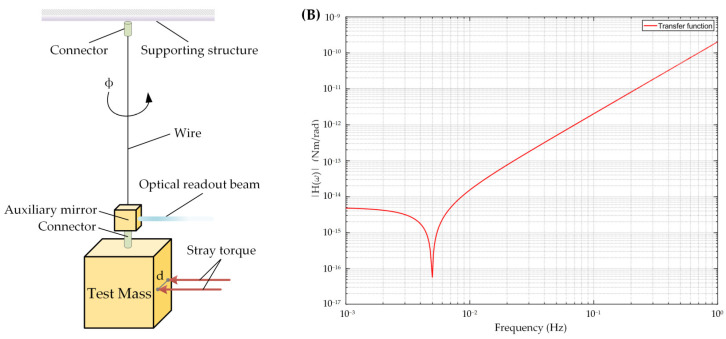
Measurement method of the TM angle using optical readout and its transfer function. (**A**) The TM suspended by a fiber is twisted under the action of external stray torque. This angle is precisely measured by an optical readout device through the auxiliary mirror. (**B**) The transfer function illustrates the relationship between torque and angle of the TM within the measurement frequency band. The resonance frequency of the pendulum is approximately 5 mHz.

Given Earth’s gravitational field, the TM’s response to forces is influenced by gravitational coupling, which is much stronger than the stray forces being investigated. To address this, the ground test device must decouple from the gravitational field. The torsion pendulum, which has been in use for over 200 years [35], can meet this requirement by suspending the cuboid TM from a thin fiber that minimizes gravitational influence in the horizontal plane while aligning the TM parallel to the Earth’s gravitational field. This design allows for the detection of weak forces and helps filter seismic noise, enhancing measurement sensitivity, as shown in Figure 1A.

For the torsion pendulum, the TM’s lowest resonant frequency is typically much higher than the measurement frequency (<1 Hz), allowing it to be treated as a rigid body. The equation for the torsion under specific external forces that cause the TM’s rotation, expressed through the converted torque, is

(1) $$I\emptyset^{\prime \prime }\left(t\right)+\xi \emptyset^{\prime }\left(t\right)+\Gamma \emptyset \left(t\right)=T\left(t\right)$$

where I is the moment of inertia of the inertial unit (all components suspended on the fiber, including the TM), ξ is the energy dissipation term, Γ is the torsional stiffness of the fiber, T(t) is the torque under external force, and ∅(t) is the rotation angle resulting from T(t).

Additionally, the torsional stiffness Γ of the fiber, determined by its physical properties, is given by the following expression:

(2) $$\Gamma =\frac{\pi R^{4}}{2L}(F+\frac{\mathrm{mg}}{\pi R^{2}})$$

Here, L, F, and R are the length, shear modulus, and radius of the fiber, respectively; m is the total mass of the inertial unit; and g is the gravitational acceleration.

Converting Formula (1) to the frequency domain via a Fourier transform, the relationship between the applied torque T(t) and the angular displacement ∅(t) can be expressed as

(3) $$T(\omega )=(\Gamma \left(1+i\delta \right)-I\omega^{2})\cdot \emptyset \left(\omega \right)$$

where ω is the angular frequency, the energy dissipation coefficient δ = 1/Q, and Q is the quality factor of the fiber. Then, the transfer function is written as

(4) $$H\left(\omega \right)=\Gamma \left(1-{\left(\frac{\omega }{\omega_{0}}\right)}^{2}+\frac{i}{Q}\right)$$

$\omega_{0}$ is the natural resonant angular frequency of the pendulum, a constant given by $\omega_{0}=\sqrt{\Gamma /I}$. Figure 1B illustrates the transfer function curve of the torsion pendulum. The relevant parameters are described in more detail in the following sections. In the frequency band below 5 mHz, the curve remains relatively flat at first. However, as it approaches 5 mHz, it shows a distinct downward trend. This behavior enhances the sensitivity of the torsion pendulum to weak forces. This increased sensitivity is advantageous for detecting subtle changes and small forces, making the pendulum particularly valuable for precision measurements. Conversely, in the frequency band above 5 mHz, the trend shifts upward, indicating that accurately describing the torque with the same magnitude requires a higher angular measurement resolution. Therefore, careful design considerations are essential to maximize the performance of the torsion pendulum.

When an external disturbance affects the torsion pendulum at a particular angular frequency, the resulting torque induces an angular displacement at the same frequency. This angular shift can be precisely measured to determine the disturbance’s magnitude.

The resolution limit of the torsion pendulum is characterized by background noise, which is influenced by thermal noise and readout noise. The torque amplitude spectrum density (ASD), $A_{\mathrm{Torq}}\left(\omega \right)$ can be expressed as

(5) $$A_{\mathrm{Torq}}(\omega )=\sqrt{S_{\mathrm{Thermal}}(\omega )+S_{\phi }(\omega )\cdot {\left|H(\omega )\right|}^{2}}$$

Here, $S_{\mathrm{Thermal}}\left(\omega \right)$ represents the thermal noise power spectral density (PSD) that arises from fiber dissipation [36]. This intrinsic noise cannot be eliminated but can be reduced through design. Its fundamental physical description is given by

(6) $$S_{\mathrm{Thermal}}\left(\omega \right)=4k_{B}T\frac{\Gamma }{\omega Q}$$

where k_B_ = 1.38 × 10^−23^(J/K) is the Boltzmann constant, T is the operating temperature, and the noise is influenced by the inertia mass, fiber length, fiber diameter, and the quality factor (Q) of the fiber.

Additionally, $S_{\phi }\left(\omega \right)$ represents the PSD of readout noise, which is critical for measurement sensitivity. This noise directly affects the accuracy of the angle readout device and can only be mitigated by enhancing measurement precision and optimizing environmental conditions. As discussed in Section 1, optical readout methods are currently the predominant approach in such scenarios.

To improve the sensitivity of the torsion pendulum, it is essential to focus on both reducing thermal noise and enhancing readout accuracy. Based on the specific implementation conditions, we will conduct a series of detailed designs to suppress thermal noise while selecting an appropriate optical readout device to accurately measure the rotation angle of the TM. This will be further elaborated upon in the subsequent section.

## 3. System Structural Construction and Performance Evaluation

Building on the basic principles of the torsion pendulum discussed above, effective noise suppression is closely tied to the system’s design. The design must not only mitigate noise limitations but also accommodate the characteristics of the IS and the test conditions. Key design components include the TM, electrode housing (EH), suspension device, vacuum system, and other related elements.

### 3.1. The Test Mass and Electrode Housing

The TM serves as the inertial element of the torsion pendulum and is a key component of the IS, acting as the inertial reference for space gravitational wave detection. It operates in a multi-physical environment, where the various types of noise listed in Section 1 will significantly degrade its performance [17,36]. As such, the design of the TM is crucial to system performance.

**Figure 2:**
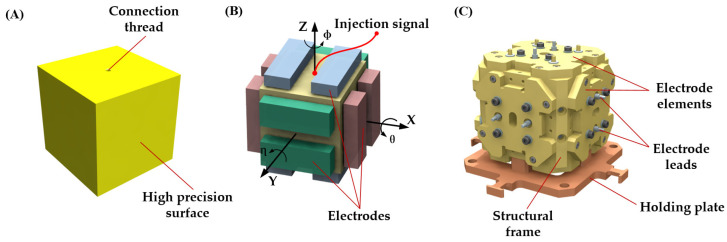
Design form of the TM and EH, along with the electrode distribution used in the torsion pendulum. (**A**) All surfaces of the TM were ultra-precision machined, with a threaded connection provided in the Z direction. (**B**) Electrode distribution in different directions: two pairs of electrodes in the same direction controlled one translational and one rotational degree of freedom, respectively. (**C**) The assembled EH, featuring electrode leads for signal transmission.

Inspired by the pioneering efforts of LISA and considering the aforementioned design constraints and future engineering needs [37], we selected a 46 mm side cube for the TM, similar to the structural configuration of the Taiji program, as illustrated in Figure 2A. Unlike the high-density alloy used in the Taiji program, the TM used in the torsion pendulum was constructed from 6061-T6 aluminum alloy. This material was chosen for its low density, low magnetic susceptibility, and high thermal conductivity. The TM had a mass of 263 g. Ultra-precision optical machining of the TM achieved a parallelism and verticality error of just 10 arcseconds between surfaces, effectively minimizing the axial coupling of stray forces. A threaded hole at the center facilitated assembly, and the entire surface was gold-coated to ensure consistent surface characteristics in preparation for charge-related noise evaluation [38].

Displacement measurement and control of the TM for space gravitational wave detection were accomplished using a capacitive sensor. The mechanical hardware of this sensor was the EH, which created differential capacitance around the TM. Since it constituted the surrounding environment of the TM and many performance metrics of the IS relied on the capacitive sensor for measurements, integrating the EH into the torsion pendulum was essential. A specialized EH assembly was developed for the Taiji program. The electrodes on all three axes had the same area of 40 mm × 15 mm, with a total of 12 electrodes evenly distributed on each axis. The gap between each electrode and the TM was 1 mm. This design featured a symmetrical cladding structure that allowed for independent control of the TM’s six DOFs across three axes, employing pairs of electrode plates for capacitive sensing in each direction, thereby enabling precise translational and rotational control, as illustrated in Figure 2B,C.

The TM was suspended within the EH using a suspension structure, which will be detailed in the following section. A dedicated front-end electronics unit injected a high-frequency AC voltage signal to the TM via the fiber. To ensure consistent thermal expansion between the TM and EH, we utilized an aluminum alloy identical to that of the TM for the base structure. Additionally, the surfaces of the EH were gold-coated to maintain uniform electric field characteristics during capacitive sensing.

### 3.2. Suspension Structure of the Test Mass

**Figure 3:**
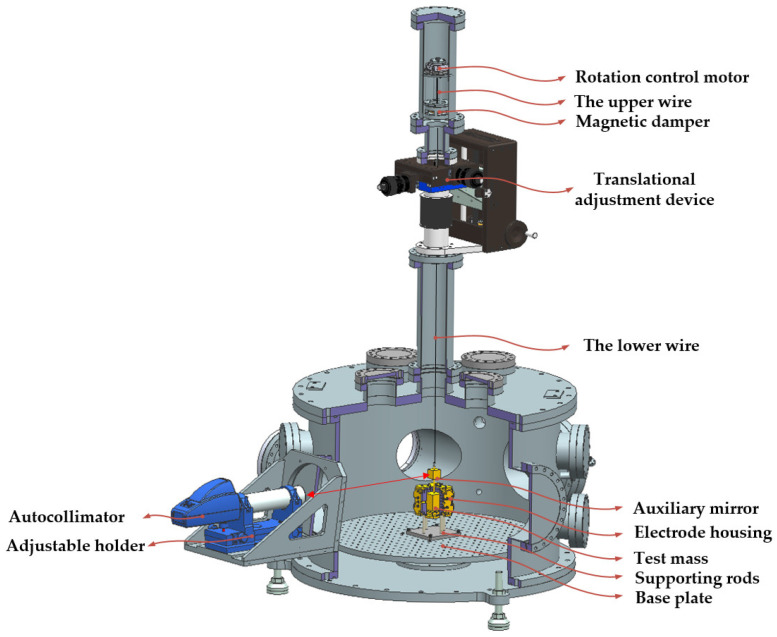
The suspension structure of the TM. The TM was suspended by two fibers separated by a magnetic damper and placed in the EH, which was mounted on the base plate. The suspension path was also provided with two sets of adjusting devices for the position adjustment of the TM.

To streamline the system, we opted for a single TM suspension solution to enhance integration. The complete suspension structure of the TM is shown in Figure 3.

To simplify implementation, we selected the commercial autocollimator TriAngle™ as the optical readout element, which has a measurement accuracy of 0.02 arcseconds, a field of view (FOV) size of 3000 arcseconds by 1920 arcseconds, and a two-DOF adjustable holder. Given the FOV limitations of the optical readout device, it was essential to align the TM with the EH at the center. To achieve this, we incorporated X, Y, Z, and θ DOFs for adjustment, which were implemented through two separate mechanisms. The linear DOFs (X, Y, Z) were adjusted using a displacement mechanism within the vacuum system. This mechanism employed a precision thread and spring for 25 mm strokes in the X and Y directions, and a worm gear with bellows for Z, allowing for 100 mm of travel. This mechanism achieved a resolution of better than 2 μm in all linear directions. The rotational DOF (θ) was provided by a top-mounted piezoelectric motor capable of continuous rotation with a resolution of 17 nrad.

In addition to alignment requirements, the system faced challenges from other types of motion during the integration process. This was particularly true for the thin and long suspension fibers, which could easily store deformation energy due to torsion. When the TM was suspended, this stored energy was released, producing not only torsional motion but also the possibility of single pendulum oscillations. The oscillation could be easily excited by the surrounding environmental fluctuations and, if not adequately controlled, lead to large amplitude motions, adversely affecting measurement accuracy. To mitigate this issue, we designed a vortex damper consisting of a damping mass positioned in a cavity formed by two symmetrically mounted magnet rings. This setup increased energy dissipation during pendulum motion, effectively suppressing unwanted oscillations. At the same time, due to the low stiffness of the fiber suspending the damping mass, it remained highly sensitive in the rotational direction. The damping mass was attached to two suspension fibers using epoxy bonding. Considering thermal noise and the strength limitations of the fiber, we selected high-elastic modulus, 99.95% pure tungsten fiber from Goodfellow^®^. The upper fiber was 120 mm long and 150 μm in diameter, while the lower fiber was 1 m long and 50 μm in diameter. After appropriate heat treatment, the Q of the system could reach 2300.

The top of the upper fiber was connected to the rotating motor, while the bottom of the lower fiber was attached to the TM using different fittings. The auxiliary mirror was fixed just above the TM to reflect the measuring light from the autocollimator. The TM was housed within the EH, while the EH was securely fixed to the vacuum system with four support rods and a base plate. Through the joint adjustment of the autocollimator and the suspension mechanism, the rotating path of the auxiliary mirror was aligned symmetrically to the center of the autocollimator target plane.

### 3.3. Vacuum System

**Figure 4:**
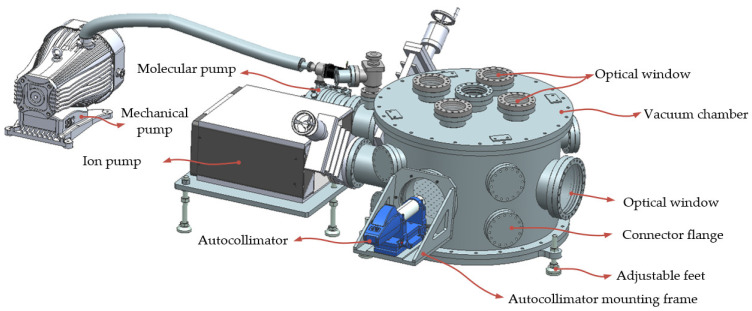
The vacuum maintenance system of the torsion pendulum. This mainly consisted of a chamber and a multistage pump group. The autocollimator was fixed on the chamber by a specially designed mounting frame to achieve the angle surveying of the TM inside.

In missions such as space gravitational wave detection, disturbances caused by gas molecules often exceed acceptable thresholds, significantly impeding relevant measurements [18]. To address this challenge, we also developed a vacuum system to enclose the TM and EH, as shown in Figure 4, thereby reducing the impact of noise on the measurements. This system consisted of a pump group, a vacuum chamber, and a monitoring and control device. The pump group utilized a dry pump and a molecular pump to progressively enhance the vacuum within the chamber, supplemented by an ion pump to maintain high vacuum levels in confined spaces. The internal vacuum could reach approximately 5 × 10^−5^ Pa. The monitoring and control device continuously tracked the internal vacuum in real time and managed the operation of each component.

The vacuum chamber was divided into upper and lower sections, connected via a flange. The upper chamber supported the suspension structure described in Section 3.2, while the lower chamber was a cylindrical cavity with a diameter of 1.2 m and a height of 0.8 m. Three adjustable feet were positioned around the bottom plate to ensure the apparatus remained level. An installation plate inside the lower chamber secured the EH and allowed for future functional expansion. The autocollimator was mounted on the edge of the optical window flange on the side of the lower chamber using a dedicated mounting frame. In addition to the optical windows, the lower chamber also featured a pass-through flange for optical or electronic connectors to support the comprehensive testing requirements of the IS. Table 1 presents the design parameters chosen for the system, aimed at enhancing its desired sensitivity.

### 3.4. Sensitivity Estimation

**Figure 5:**
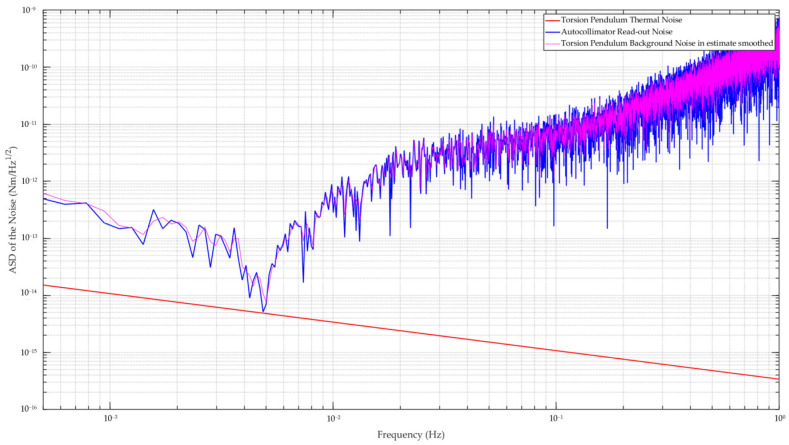
Main noise curves associated with the designed torsion pendulum. The red curve represents the thermal noise of the torsion pendulum, while the blue curve illustrates the autocollimator background noise measured during the experiment. The carmine curve represents the estimated background noise of the torsion pendulum, obtained by integrating the thermal noise and autocollimator readout noise; it nearly coincided with the blue curve.

Section 2 discusses the major factors influencing the sensitivity of the torsion pendulum, specifically thermal noise and readout noise. Thermal noise, which is constrained by physical limits, plays an important role in determining sensitivity. However, its impact is closely related to structural design. The design parameters presented earlier were tailored to meet the current requirements. By synthesizing Formula (6) with the structural design parameters outlined in Table 1, we derived the thermal noise curve for the torsion pendulum, shown by the red line in Figure 5. This curve indicated that the sensitivity limit of the torsion pendulum ranged from approximately 4 × 10^−16^ Nm/Hz^1/2^ to 1 × 10^−15^ Nm/Hz^1/2^ across a frequency range from 1 mHz to 0.1 Hz. It demonstrated a decreasing trend across the entire frequency band, with thermal noise diminishing as frequency increased.

To assess the optical readout noise, we conducted tests by establishing a stable optical path with a rigid connection, using the autocollimator and auxiliary mirror from the previously described design. The tests were performed in the same environment as the subsequent torsional pendulum apparatus experiment. The autocollimator operated at a sampling frequency of 10 Hz. After approximately 18 h of measurement and sampling, we selected a stable, long-term dataset and applied Formula (3) to convert the data into torque signals, represented by the blue line in Figure 5. The results indicated that the readout noise was nearly one order of magnitude greater than the thermal noise across the entire measurement range. This suggested that optical readout noise had a greater influence on the final sensitivity of the torsion pendulum than thermal noise.

Building upon this analysis, we used Formula (5) to estimate the comprehensive sensitivity of the designed torsion pendulum, yielding the carmine curve shown in Figure 5 after two-times smoothing. This curve closely aligned with the optical readout noise, further confirming that the sensitivity of the apparatus was primarily governed by the performance of the autocollimator. Consequently, we concluded that the best achievable sensitivity of the current torsion pendulum was approximately 7.7 × 10^−15^ Nm/Hz^1/2^ at 5 mHz, and the sensitivity of the device was expected to reach 1.0 × 10^−11^ Nm/Hz^1/2^ across the entire measurement frequency range below 0.1 Hz.

## 4. Background Noise Experiments

**Figure 6:**
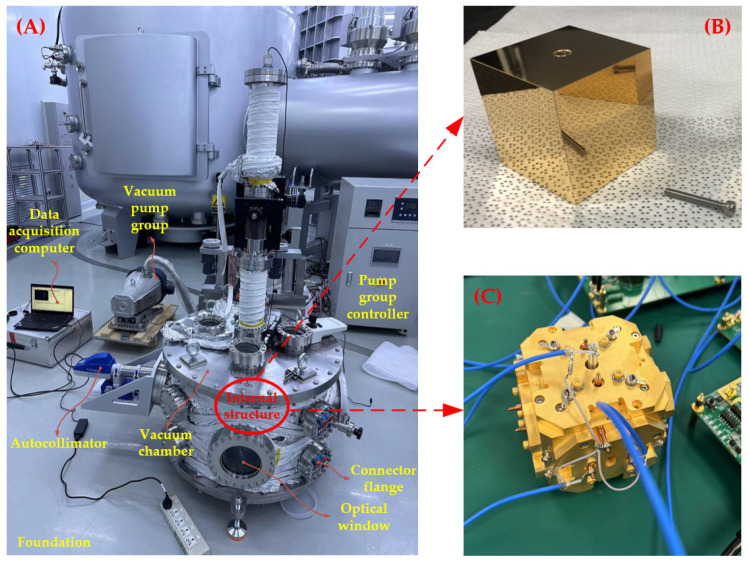
Integrated torsion pendulum as well as the internal TM and EH units. (**A**) The whole apparatus under experimentation. (**B**) The TM before integration. (**C**) The electrode housing under electronics testing.

After completing the system analysis and detailed design of the components, we assembled the torsion pendulum in the laboratory of the Center for Gravitational Wave Experiment at the Institute of Mechanics, Chinese Academy of Sciences. To minimize contamination risks, the entire system integration was conducted in a Class 10,000 cleanroom. Additionally, to reduce crosstalk from ambient vibrations, we placed the apparatus on a separate foundation within the laboratory, as illustrated in Figure 6. By adjusting the rotating motor at the top and the adjustable holder of the autocollimator, the torsion angle range of the TM accounted for less than 1/3 of the autocollimator’s measuring range, while the vacuum in the chamber could be maintained up to 1.8 × 10^−5^ Pa. This setup allowed us to achieve the basic measuring functionality of the system.

**Figure 7:**
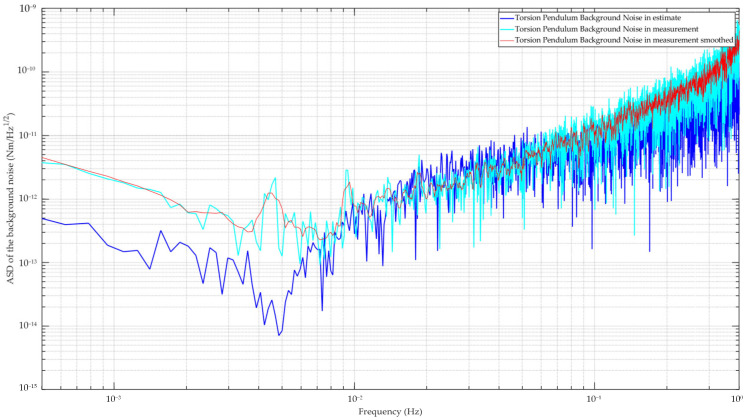
The background noise curves of the torsion pendulum system. The blue line represents the estimated background noise as detailed in Section 3.4. The cyan line depicts the actual measured background noise recorded from the apparatus during experiments. The red line illustrates a smoothed version of the background noise data from the torsion pendulum.

Under these conditions, we performed measurement experiments to assess the torsion pendulum’s background torque noise and evaluate its sensitivity across the frequency range. To minimize interference, these experiments were scheduled during nights or weekends. Despite this, temperature fluctuations of approximately 10 K occurred throughout the day, and the maximum seismic noise was recorded at 1 × 10^−6^ m/s^2^/Hz^1/2^. After several rounds of data acquisition and processing, we compared the measured sensitivity with the predicted sensitivity curve from Section 3.4, as shown in Figure 7.

The experimental results aligned closely with the predicted values, with discrepancies observed above 10 mHz. At frequencies below 10 mHz, the measured sensitivity exhibited a degradation of about one order of magnitude. These variations may have been largely due to temperature fluctuations, which changed by several degrees during the experiment. Seismic and other environmental noise likely contributed to the observed discrepancies. Furthermore, the measurement curve exhibited peaks at several frequency points, corresponding to 4.5 mHz and its harmonic frequencies. It is likely that this was influenced by the signal aliasing phenomenon. Nevertheless, the overall sensitivity remained better than 1 × 10^−11^ Nm/Hz^1/2^ below 0.1 Hz, with a notable sensitivity of 2.2 × 10^−13^ Nm/Hz^1/2^ at 7 mHz. The performance met our current measurement requirements of 3 × 10^−9^ m/s^2^/Hz^1/2^ at 10 mHz for low-orbit science missions.

## 5. Conclusions

In this paper, we introduced a torsion pendulum system designed for IS testing. The primary objective of this apparatus was to determine the upper limit of weak stray force noise affecting the TM and verify various noise models.

The analysis of the fundamental principles of the torsion pendulum identified thermal noise and readout noise as the main factors affecting the sensitivity. Thermal noise was determined by the structural parameters of the system and could not be eliminated. To reduce the effect of thermal noise, a series of related components were designed and developed based on the single TM. For readout noise, a commercial autocollimator was used for its excellent angle measurement ability and assembly convenience. After comprehensive considerations of noise suppression, integration testing, functional expansion, and engineering implementation, we designed and developed the TM, electrode housing, suspension structure of the TM, vacuum maintenance system, and various other elements. The sensitivity of the torsion pendulum was estimated based on the thermal noise and autocollimator background noise.

After careful integration and testing, the apparatus conducted a series of background noise tests. The results demonstrate that the torsion pendulum system achieves a sensitivity better than 1 × 10^−11^ Nm/Hz^1/2^ below 0.1 Hz, with sensitivity reaching 2.2 × 10^−13^ Nm/Hz^1/2^ near 7 mHz. This performance is sufficient for low-orbit science missions at this stage.

For future space gravitational wave detection or other applications requiring higher precision, there remains a gap in the sensitivity of the torsion pendulum system developed above. However, these gaps can be narrowed through targeted system upgrades, which are currently underway. The planned advancements include the following:

1. Integrated Heterodyne Interference: The design of an integrated heterodyne interference laser measurement system to replace the commercial autocollimator [39], effectively suppressing readout noise while improving measurement accuracy, particularly in the low-frequency range.
2. Enhanced Vibration Isolation: The implementation of an advanced vibration isolation system to further mitigate the effects of seismic noise [33].
3. Temperature Control and Electromagnetic Shielding: The addition of system-level temperature control and electromagnetic shielding devices to minimize crosstalk from internal and external environments.
4. Different structural forms: The development of multistage or multi-mass torsion pendulum systems for increasing sensitivity [28,31], which will also facilitate the direct acquisition of acceleration data, making them more suitable for future applications.
5. Further Exploration into Signal Acquisition and Processing: The utilization of anti-aliasing and digital filtering strategies, which will significantly improve the precision of the sampled signal. Optimizing the sampling frequency and implementing appropriate extraction or interpolation procedures will further enhance signal quality. Additionally, developing environmental noise extraction and subtraction technologies will strengthen the stability and reliability of the measurement.

These advancements will significantly enhance the measurement capabilities of the torsion pendulum system. The apparatus will be employed in a series of experiments, including noise limit determination, the validation of different noise models, and the evaluation of coupling errors, among others. These experiments will support the development of ISs for the Taiji program and other related scientific missions.

## Figures and Tables

**Table 1:** Basic parameters of the torsion pendulum.

	Parameters	Values	Units	Comment
Test mass	d	46	mm	Length of sides
	m_total_	0.238	kg	Total mass of inertial unit
Tungsten fiber	L	1	m	Length of lower fiber
	R	50	μm	Radius of lower fiber
	G	161	GPa	Shear modulus
	Q	2300	/	Quality factor
Environment condition	g	9.801	m/s^2^	Gravitational acceleration
	T	300	K	Operating temperature

## Data Availability

Data are contained within the article.

## References

[1] Abbott B.P., Abbott R., Abbott T.D., Abernathy M.R., Acernese F., Ackley K., Adams C., Adams T., Addesso P., Adhikari R.X. (2016). GW150914: The Advanced LIGO detectors in the era of first discoveries. Phys. Rev. Lett..

[2] Hendry M. (2007). An Introduction to General Relativity, Gravitational Waves and Detection Principles.

[3] Ezquiaga J.M., Zumalacárregui M. (2018). Dark energy in light of multi-messenger gravitational-wave astronomy. Front. Astron. Space Sci..

[4] Riles K. (2013). Gravitational waves: Sources, detectors and searches. Prog. Part. Nucl. Phys..

[5] Amaro-Seoane P., Audley H., Babak S., Baker J., Barausse E., Bender P., Berti E., Binetruy P., Born M., Bortoluzzi D. (2017). Laser interferometer space antenna. arXiv.

[6] Anderson G., Anderson J., Anderson M., Aveni G., Bame D., Barela P., Blackman K., Carmain A., Chen L., Armano M. (2018). Experimental results from the ST7 mission on LISA Pathfinder. Phys. Rev. D.

[7] Armano M., Audley H., Baird J., Binetruy P., Born M., Bortoluzzi D., Castelli E., Cavalleri A., Cesarini A., Chiavegato V. (2024). In-depth analysis of LISA Pathfinder performance results: Time evolution, noise projection, physical models, and implications for LISA. Phys. Rev. D.

[8] Harry G.M., Fritschel P., Shaddock D.A., Folkner W., Phinney E.S. (2006). Laser interferometry for the big bang observer. Class. Quantum Gravity.

[9] Kawamura S., Nakamura T., Ando M., Tsubono K., Tanaka T., Funaki I., Seto N., Numata K., Sato S., Ioka K. (2006). The Japanese space gravitational wave antenna—DECIGO. Class. Quantum Gravity.

[10] Ni W.T. (2013). ASTROD-GW: Overview and progress. Int. J. Mod. Phys. D.

[11] Wu Y.L. (2018). Hyperunified field theory and Taiji program in space for GWD. Int. J. Mod. Phys. A.

[12] Luo J., Chen L.S., Duan H.Z., Gong Y.-G., Hu S., Ji J., Liu Q., Mei J., Milyukov V., Sazhin M. (2016). TianQin: A space-borne gravitational wave detector. Class. Quantum Gravity.

[13] Hu W.R., Wu Y.L. (2017). The Taiji Program in Space for gravitational wave physics and the nature of gravity. Natl. Sci. Rev..

[14] Luo Z., Min Z., Jin G., Wu Y., Hu W. (2020). Introduction of Chinese space-borne gravitational wave detection program “Taiji” and “Taiji-1” satellite mission. J. Deep. Space Explor..

[15] Luo Z., Wang Y., Wu Y., Hu W.R., Jin G. (2021). The Taiji program: A concise overview. Prog. Theor. Exp. Phys..

[16] Armano M., Audley H., Auger G., Baird J., Binetruy P., Born M., Bortoluzzi D., Brandt N., Bursi A., Caleno M. (2015). The LISA pathfinder mission. J. Phys. Conf. Ser..

[17] Cavalleri A., Ciani G., Dolesi R., Hueller M., Nicolodi D., Tombolato D., Wass P.J., Weber W.J., Vitale S., Carbone L. (2009). Direct force measurements for testing the LISA Pathfinder gravitational reference sensor. Class. Quantum Gravity.

[18] Weber W.J., Bortoluzzi D., Bosetti P., Consolini G., Dolesi R., Vitale S. (2022). Application of LISA gravitational reference sensor hardware to future intersatellite geodesy missions. Remote Sens..

[19] Schumaker B.L. (2003). Disturbance reduction requirements for LISA. Class. Quantum Gravity.

[20] Luo J., Lei Y., Shao C., Liu J., Li D., Liu R., Li Q. (2020). Scheme of G measurement with large amplitude torsion pendulum. Phys. Rev. D.

[21] Wang H., Wang W., Yan J., Fu C., Liu W. (2022). Measurement and prediction of micronewton class thrust of electric propulsion based on the torsional pendulum and machine learning technique. IEEE Trans. Instrum. Meas..

[22] Wagner T.A., Schlamminger S., Gundlach J.H., Adelberger E.G. (2012). Torsion-balance tests of the weak equivalence principle. Class. Quantum Gravity.

[23] Russano G., Cavalleri A., Cesarini A., Dolesi R., Ferroni V., Gibert F., Weber W.J. (2018). Measuring fN force variations in the presence of constant nN forces: A torsion pendulum ground test of the LISA Pathfinder free-fall mode. Class. Quantum Gravity.

[24] Cavalleri A., Ciani G., Dolesi R., Heptonstall A., Hueller M., Nicolodi D., Rowan S., Tombolato D., Vitale S., Wass P.J. (2009). A new torsion pendulum for testing the limits of free-fall for LISA TMes. Class. Quantum Gravity.

[25] Ciani G. (2008). Free-Fall of LISA Test Masses: A New Torsion Pendulum to Test Translational Acceleration. Ph.D. Thesis.

[26] Marconi L., Stanga R., Lorenzini M., Grimani C., Bassan M., Pucacco G., Di Fiore L., De Rosa R., Garufi F., Milano L. (2010). The 2 Degrees of Freedom facility in Firenze for the study of weak forces. J. Phys. Conf. Ser..

[27] Bassan M., Cavalleri A., De Laurentis M., De Marchi F., De Rosa R., Di Fiore L., Dolesi R., Finetti N., Garufi F., Grado A. (2016). Approaching free fall on two degrees of freedom: Simultaneous measurement of residual force and torque on a double torsion pendulum. Phys. Rev. Lett..

[28] Apple S.M., Chilton A., Olatunde T., Bickerstaff B., Hillsberry D., Parry S., Ciani G., Mueller G., Conklin J. University of Florida Torsion Pendulum for Testing Key LISA Technology. Proceedings of the AIAA SPACE and Astronautics Forum and Exposition.

[29] Chilton A., Shelley R., Olatunde T., Ciani G., Conklin J.W., Mueller G. (2015). The UF Torsion Pendulum, a LISA Technology Testbed: Sensing System and Initial Results. J. Phys. Conf. Ser..

[30] Yang F., Bai Y., Hong W., Li H., Liu L., Sumner T.J., Zhou Z. (2020). Investigation of charge management using UV LED device with a torsion pendulum for TianQin. Class. Quantum Gravity.

[31] Bai Y., Li Z., Hu M., Liu L., Qu S., Tan D., Zhou Z. (2017). Research and development of electrostatic accelerometers for space science missions at HUST. Sensors.

[32] Ross M.P., Venkateswara K., Hagedorn C.A., Leupold C.J., Forsyth P.W.F., Wegner J.D., Shaw E.A., Lee J.G., Gundlach J.H. (2021). A low-frequency torsion pendulum with interferometric readout. Rev. Sci. Instrum..

[33] Bergmann G., Cordes C., Gentemann C., Händchen V., Qinglan W., Yan H., Danzmann K., Heinzel G., Mehmet M. (2024). A torsion balance as a weak-force testbed for novel optical inertial sensors. Class. Quantum Gravity.

[34] Luo Z., Guo Z.K., Jin G., Wu Y., Hu W. (2020). A brief analysis to Taiji: Science and technology. Results Phys..

[35] Martínez A.A. (2006). Replication of Coulomb’s torsion balance experiment. Arch. Hist. Exact Sci..

[36] González G.I., Saulson P.R. (1995). Brownian motion of a torsion pendulum with internal friction. Phys. Lett. A.

[37] Han R., Cai M., Yang T., Xu L., Xia Q., Jia X. (2024). Study on Test-Mass Charging for Taiji Gravitational Wave Observatory. Space Weather.

[38] Inchauspé H., Olatunde T., Apple S., Parry S., Letson B., Turetta N., Mueller G., Wass P.J., Conklin J.W. (2020). Numerical modeling and experimental demonstration of pulsed charge control for the space inertial sensor used in LISA. Phys. Rev. D.

[39] Wang S., Liu H., Dai L., Luo Z., Xu P., Li P., Gao R., Li D., Qi K. (2023). Using DWS Optical Readout to Improve the Sensitivity of Torsion Pendulum. Sensors.

